# Model Organisms in the Fight against Muscular Dystrophy: Lessons from *Drosophila* and Zebrafish

**DOI:** 10.3390/molecules20046237

**Published:** 2015-04-09

**Authors:** Emilie Plantié, Marta Migocka-Patrzałek, Małgorzata Daczewska, Krzysztof Jagla

**Affiliations:** 1 GReD (Genetics, Reproduction and Development laboratory), INSERM U1103, CNRS UMR6293, University of Clermont-Ferrand, 28 place Henri-Dunant, 63000 Clermont-Ferrand, France; E-Mail: emilie.plantie@udamail.fr; 2 Department of Animal Developmental Biology, Institute of Experimental Biology, University of Wroclaw, 21 Sienkiewicza Street, 50-335 Wroclaw, Poland; E-Mails: marta.migocka-patrzalek@uni.wroc.pl (M.M.-P.); malgorzata.daczewska@uni.wroc.pl (M.D.)

**Keywords:** muscular dystrophies, animal models, *Drosophila*, zebrafish, pathophysiology, therapeutic screens

## Abstract

Muscular dystrophies (MD) are a heterogeneous group of genetic disorders that cause muscle weakness, abnormal contractions and muscle wasting, often leading to premature death. More than 30 types of MD have been described so far; those most thoroughly studied are Duchenne muscular dystrophy (DMD), myotonic dystrophy type 1 (DM1) and congenital MDs. Structurally, physiologically and biochemically, MDs affect different types of muscles and cause individual symptoms such that genetic and molecular pathways underlying their pathogenesis thus remain poorly understood. To improve our knowledge of how MD-caused muscle defects arise and to find efficacious therapeutic treatments, different animal models have been generated and applied. Among these, simple non-mammalian *Drosophila* and zebrafish models have proved most useful. This review discusses how zebrafish and *Drosophila* MD have helped to identify genetic determinants of MDs and design innovative therapeutic strategies with a special focus on DMD, DM1 and congenital MDs.

## 1. Introduction

Muscular dystrophies (MDs) are a heterogeneous group of inherited rare disorders primarily affecting the muscle system, causing progressive skeletal muscle weakness, and often associated with cardiomyopathy and respiratory problems [1,2]. Duchenne MD (DMD), Becker MD (BMD), Limb-Girdle MD (LGMD), myotonic dystrophy type 1 (DM1), oculo-pharyngeal MD (OPMD), fascio-scapulo-humeral MD (FSHMD), Emery-Dreifuss MD (EDMD), distal muscular dystrophy (DD) and congenital MD (CMD) are the nine most common muscular dystrophies. Among them, DMD caused by the mutation in the *Dystrophin* gene identified more than twenty years ago [3,4,5] still has no effective therapy, illustrating a lasting need to find efficacious treatments for MDs.

Due to technical and ethical reasons, it is not always possible to investigate disease processes directly in humans. Model organisms, both mammalian and non-mammalian, are essential to understanding normal and pathological muscle development in humans. The major non-mammalian animal models, the fruitfly *Drosophila melanogaster*, the zebrafish *Danio rerio* and the nematode *Caenorhabditis elegans*, are considered useful alternatives for studying human diseases and particularly muscular dystrophies [3]. Among them, *Drosophila melanogaster* has been used as a model organism for over 100 years, particularly in genetics, where it was useful to better understand the concept of trait heritability [4,5]. The fruitfly genome was fully sequenced in 2000 [6], revealing that key physiological processes and disease-related genes are well-conserved between flies and humans [7,8], making it a model of choice for these studies. Finally, the availability of *Drosophila* transgenic and mutant line collections facilitates functional analyses of disease genes. Compared with the fruitfly, the worm, owing to a lack of muscle cell fusion, restricted gene conservation and a short lifespan, appears suitable for studying only some aspects of muscle defects observed in MDs. 

By contrast, the vertebrate model zebrafish conserves the majority (84%) of the genes known to be associated with human diseases, both rare and common [9], with significant degrees of synteny between the conserved genes [10]. The zebrafish offers many advantages: it can quickly produce a large number of externally developing, diploid embryos; the transparency of zebrafish embryos and larvae allows real-time imaging of all developmental stages; and the genome sequence, as for *Drosophila*, is available [9]. To generate disease models, three main methods are used in zebrafish: morpholino-mediated knockdown [11,12]; *N*-ethyl-*N*-nitrosourea (ENU) mutagenesis [13], which is widely used to identify new genes involved in muscles diseases [14,15,16,17]; and finally genome-editing technologies such as the clustered regularly interspaced short palindromic repeats technique (CRISPR), successfully used in both zebrafish [18,19,20] and *Drosophila* [21,22].

Importantly, besides gene conservation, *Drosophila* and zebrafish muscles share many structural, histological and functional similarities with human muscle [23,24,25]. These similarities further support the view that *Drosophila* and zebrafish models are well-suited to studying human muscle disorders including MDs.

## 2. *Drosophila* and Zebrafish Models in Understanding the Molecular Basis of Muscular Diseases 

*Drosophila* and zebrafish models have been used to study human diseases for many years, and have helped us gain a better understanding of the molecular mechanisms underlying these disorders, explore therapeutic strategies and restore normal conditions in affected individuals [8,26,27,28,29,30,31,32].

A growing body of new models of muscular diseases is being created using these animals.

For the purpose of this review, we will focus on some particular MDs, namely DMD [33,34], DM1 [35,36,37,38] and congenital MD (Walker-Warburg syndrome [39] and CMD Type 1 A [40]), which have been modelled in both fruitfly and zebrafish.

The most common muscular dystrophies are the X-linked, lethal childhood (more severe) DMD and the (less severe) BMD, both caused by mutations in the dystrophin gene. In skeletal muscles, dystrophin associates with various proteins to form a large membrane-bound protein complex called the dystrophin-associated glycoprotein complex (DGC). DGC plays a crucial structural role in linking the actin filaments to the extracellular matrix. Mutation of the dystrophin gene destabilizes this complex and leads to progressive fibre damage, sarcolemma destabilization and leakage [41,42]. Comparative genomic studies have shown sequence conservation of dystrophin in vertebrate and invertebrate species including zebrafish and *Drosophila* [43].

The second MD described, myotonic dystrophy type 1 (DM1), is a neuromuscular disorder caused by a microsatellite CTG repeat expansion in the 3'UTR of the dystrophia myotonica protein kinase (DMPK) gene. Mutated transcripts with expanded repeats accumulate in the nuclei and sequester an alternative splicing factor, called MBNL1, causing foci formation. It leads to splicing defects that can cause a variety of symptoms, ranging from myotonia, muscle weakness to heart symptoms such as arrhythmia or conduction defects [44,45,46,47]. 

Although DM1 is a well-characterized disorder, its molecular mechanisms are not yet fully understood [48].

The third subgroup of MDs described here are the congenital MDs, a heterogeneous group of diseases that include the dystroglycanopathies (caused by mutations in POMT1, POMT2, FKTN, FKRP and other genes), laminin alpha-2 deficiency (CMD 1A), collagen VI-deficient CMD, SEPN1-related CMD, LMNA-related CMD (L-CMD) [49,50,51,52] and the most severe congenital MD, Walker-Warburg syndrome (WWS). This rare disorder mainly affects the ocular system and the brain, but also muscles, causing muscle weakness, mental retardation and vision impairment [53]. Several genes are involved in WWS, but major mutations target POMT1 and POMT2 genes [54].

Despite the focus on these three particular subtypes of MD, others have been characterized in *Drosophila* [33,35,36,39,55,56,57,58,59] and zebrafish [32,37,38,40,60,61,62,63] and are listed in Table 1.

### 2.1. Drosophila for Disease Modelling and Understanding of Pathogenesis Mechanisms of MD

As stated above, *Drosophila* has been used for several decades as a model organism to characterize pathogenesis mechanisms of human diseases. For example, in 2002 Takeyama and co-workers developed a spinobulbar muscular atrophy (SBMA) *Drosophila* model, consisting of CAG expansion in androgen receptor (AR) gene, and showed that a ligand-dependent activation of AR is necessary for motor neuron degeneration [64]. Similarly, *smn* hypomorphic mutants [65] were found to recapitulate symptoms observed in spinal muscular atrophy (SMA), and this model helped to identify the involvement of the BMP pathway in SMA pathogenesis [66]. Among many others, *Drosophila* models were also instrumental in gaining insights into the pathogenesis of OPMD [57] and Barth syndrome [67].

**Table 1:** Major Muscular Dystrophies (MD) modelled in *Drosophila* and zebrafish.

Disease	Mutated Gene	Animal Model		Mutation Type in Animal Models		Shared Symptoms with Patients	Ref.
		*D*	Z	*Drosophila*	Zebrafish		
**DMD/BMD**	Dystrophin	✓	✓	Dys deletion mutants	Nonsense mutation in dystrophin gene	Age-dependent muscle degeneration/Loss of muscle integrity	[33,55]
					Splice mutation in dystrophin gene		[60,61,62]
**LGMD**	Lamin A/C Sarcoglycan Dysferlin POMT1	✓	✗	Partial/null mutations in the Drosophila δ-sarcoglycan locus	✗	Reduced lifespan & mobility in aged flies	[56]
**DM1**	DMPK	✓	✓	Expression of CTG repeats in adult/larval muscle	MBNL gene knockdown	Myotonia/Muscle defects Splicing defects Foci formation	[35,36]
					Injection of CUG repeat-containing mRNA		[37,38,68]
**OPMD**	PABPN1	✓	✗	Human PABPN1-17ala expressed in adult muscle	✗	Progressive muscle degeneration Nuclear inclusions	[57]
**EDMD**	Emerin Lamin A/C	✓	✗	Transgenic flies expressing a mutant form of Lamin-C (lacking the first 42 AA)	✗	Muscle defects Early death	[58,59]
**CMD**	POMT1 Fukutin Laminin α2	✓	✓	Mutants for POMT1 & POMT2 Drosophila orthologs	Point mutation in laminin α2 gene	Shortened lifespan Age-dependent severity of muscle phenotypes/Disorder of primary motor neurons innervation	[39,40]

Notes: Abbreviations: ***D** Drosophila*, **Z** Zebrafish, **DMD** Duchenne MD, **BMD** Becker MD, **LGMD** Limb-Girdle MD, **DM1** Myotonic Dystrophy Type 1, **OPMD** Oculo-Pharyngeal MD, **EDMD** Emery-Dreifuss MD, **CMD** Congenital MD. More comprehensive tables of additional models can be found in other recent reviews such as [32,63].

Regarding DMD, the conservation of genes forming the DGC complex and their reduced number makes the fly a good model to study DMD [69]. To model this disease, mutations in Dg and Dys, as well as RNAi lines against these genes, have been generated and analysed. Shcherbata *et al.* demonstrated mobility defects and age-dependent muscle degeneration and neural defects in *dys* mutants [33]. Also, the specific loss of dys in the heart led to an age-dependent disruption of the myofibrillar organization within the myocardium, and to alterations in cardiac performance and development of dilated cardiomyopathy [70]. More recently, a study of the *Drosophila* DMD model exposed to chronic hypoxia revealed impairment of the muscle function *in vivo* [55]. It has been assumed that systemic hypoxia could contribute to DMD pathophysiology in patients. Moreover, mdx mice (the naturally occurring DMD model) present worsened diaphragmatic dysfunction following hypoxia [71]. These models confirm that *Drosophila* is a suitable model to study DMD, demonstrating that Dg and Dys interaction is necessary for muscle maintenance, and that the reduced Dys level could explain the degeneration observed in patients. 

In addition to DMD, the RNA-dominant disorder DM1 has been modelled in the fruitfly by several groups. First in 2005, Houseley and colleagues showed the sequestration of the MBNL1 orthologue in muscle nuclei by CTG_162_ toxic repeats, but did not detect any locomotor activity perturbation, muscle defects or reduced lifespan [72]. Shortly afterwards, another DM1 model with 480 interrupted CUG repeats was generated, again showing the accumulation of toxic repeats in nuclear foci, colocalizing with Mbl (MBNL1 orthologue). The DM1 flies exhibited muscle wasting and degeneration, also seen in patients as a result of decreased MBNL1 and increased CUGBP1 levels [35]. More recently, our group generated novel DM1 models consisting of inducible transgenic lines carrying increasing numbers of CTG repeats, which could allow the assessment of effects of CTG repeat size. In addition to foci formation and reduced motility, we observed a hypercontracted phenotype in DM1 larvae, reminiscent of myotonia in patients. It could involve the calcium pump dSERCA, an Mbl splice target because the membrane dSERCA isoform was sufficient to rescue this DM1-induced hypercontraction phenotype in our model. Also, we demonstrated that nuclear accumulation of toxic CUG repeats can affect gene expression independently of splicing [36].

Regarding Walker-Warburg syndrome (WWS), Wairkar and co-workers performed a screen for synaptic abnormalities in *Drosophila* and identified mutations in POMT1 orthologue, *rotated abdomen* (*rt*). They demonstrated that *rt* is required to glycosylate the *Drosophila* dystroglycan ortholog Dg *in vivo*, suggesting that this defective glycosylation in *rt* mutants could impair synaptic transmission and cause the mental symptoms [73]. Muscle structure and behaviour of knockdown flies for POMT1 and POMT2 orthologues, *rt* and *twisted (tw)* respectively, have also been analysed by Ueyama *et al.* [39]. They demonstrated a reduced lifespan, together with locomotion defects and adversely affected muscle structure, also seen in WWS patients. Moreover, they described an age-related worsening of the ultrastructural abnormalities in muscles, associated with excessive apoptosis of myoblasts in the wing disc of *tw* mutants (POMT2 orthologue) [39]. The authors proposed a new mechanism caused by mutation in POMT genes, involving increased apoptosis that could explain muscle disorganization and muscle defects observed in the disease.

### 2.2. Zebrafish Models Designed to Dissect the Molecular Mechanisms of MD

Among all animal model organisms, many unique advantages of the zebrafish (described above) make it an attractive model for human diseases and for dissecting pathological processes. Zebrafish models have been used, for example, to study congenital and hereditary diseases; carcinogenesis; infection; inflammation and wound healing; immunological, metabolic, endocrine and nutritional diseases; and psychological and behavioural abnormalities, as reviewed in [29].

One of the animal models for DMD investigation are naturally isolated *mdx* mouse mutants. However, *mdx* mice have only a mild DMD phenotype owing to the high regenerative capacity of mouse muscles [74,75,76]. Another widely used model to study MD is the zebrafish. Different groups reported that the zebrafish carries an orthologue of the human dystrophin gene, encoding a 400 kDa protein that mimics the subcellular pattern of human dystrophin in adult muscles [77,78]. Large-scale mutagenesis screening in zebrafish has identified a large number of mutants that affect muscle formation, one class of which, called “dystrophic class mutants,” presented damaged and disorganized structure of muscles under polarized light. Among them, the *sapje* (*sap*) allele has been shown to carry mutation at zf-dystrophin, the zebrafish orthologue of human DMD locus [13,61,79]. This nonsense mutation resulting in a premature stop codon within the N-terminal actin-binding domain of *dystrophin* removes the large muscle-specific isoform, and causes progressive muscle degeneration by disrupting the link between the actin cytoskeleton and the extracellular matrix in skeletal muscles. Compared with the *mdx* mice, homozygous *sap* mutant embryos display more severe phenotypes, sharing a large number of similarities with human disease, e.g., humans and zebrafish lack the compensatory levels of utrophin thought to protect *mdx* mice [80,81]. In homozygous *sap* mutants, dystrophin deficiency causes a progressive loss of muscle integrity as a result of muscle attachment failure at the embryonic myotendinous junction (MTJ) [62]. It was also found that in *sap* mutants, fibre degeneration involves separation of the terminal sarcomeres from the terminal sarcolemma, as well as detachment of the terminal sarcolemma from the myoseptum [82]. Furthermore, myofibrils in *sap* mutants shown by transmission electron microscopy exhibit nuclear condensation indicating cell death [61].

Numerous animal models of DM1 have already been generated, but they all have some limitations. Making use of the advantages of zebrafish, Machuca-Tzili and co-workers applied this model to determine the role of MBNL in DM1 pathogenesis. The morpholino knockdown model showed the essential role of the MBNL2 gene during embryonic development. A lack of *mbnl2* caused muscle defects and splicing abnormalities, typical of DM1. An absence of the *mbnl2* gene also led to disruption of myofibril organization in skeletal and heart muscles, and reduction of the amount of slow and fast muscles. The phenotypes of *mbnl2*-morphants were specifically due to *mbnl2* knockdown and were rescued with *mbnl2* mRNA [37]. More recently, Todd *et al.* described a new zebrafish model designed to explore the impact of CUG repeat expression during early development. This model of DM1 is based on injection of mRNA, carrying expanded CUG repeats, at a single-cell stage zebrafish embryo. This leads to alteration in fish morphology, behaviour and transcriptional activity during early development. CUG repeat-containing mRNA degrades progressively and is no longer detected after 72 hpf; embryos that survive the first 48 h thus show no significant alterations as adult fish. As stated above, the sequestration of MBNL proteins by CUG RNA is an important pathogenic mechanism of DM. The authors showed that coexpression of zebrafish *mbnl2* and CUG RNA suppresses the CUG repeats-mediated toxicity. Thus these studies reveal the influence of toxic CUG repeat-containing mRNA during early development, and suggest that zebrafish DM1 models are attractive tools to conduct small-molecule screening and develop new drug therapies for myotonic dystrophy [38]. The zebrafish DM1 model described above was applied to study the *in vitro* effects of CUG pseudouridylation [68]. Pseudouridine is an isomerization product of uridine and is the most common structure-stabilizing RNA modification. The studies showed that pseudouridylation of CUG repeats prevents CUG RNA toxicity in the zebrafish DM1 model, implying that the structure of toxic RNAs plays a significant role in the pathogenesis of DM1. These results open new perspectives for the understanding of disease mechanisms and for corresponding drug design. 

Zebrafish is also an excellent animal model to study congenital muscular dystrophies including CMD Type 1 A (CMD 1A) caused by mutation in the human laminin α2 (*LAMA2*) gene [83]. The *LAMA2* mutation leads to muscle degeneration, necrosis, fibrosis and abnormalities in peripheral nerves. *LAMA2* plays a crucial structural role in the maintenance of sarcolemmal integrity through its interaction with dystrophin-associated glycoprotein. Hall and co-workers [40] revealed that zebrafish dystrophic mutant *candyfloss* is the result of mutations in the laminin α2 (*LAMA2*) gene. The authors demonstrated that early muscle differentiation and myoblast fusion occurred correctly, indicating that deficiency in early Lama2 signalling does not contribute to muscle pathology. However, they observed that innervation by the primary motor neurons was affected. The authors find that muscle tissue damage and subsequent cell death occur through mechanically induced fibre detachments in the absence of sarcolemmal rupture.

## 3. Major Advances for MD through Therapeutic Drug Screening in the Fruitfly and Zebrafish

In this section, fundamental findings in *Drosophila* and zebrafish models to help cure muscular dystrophies will be discussed. We will focus on drug and genetic screening performed in DMD and DM1 models in both species (Table 2).

**Table 2:** Screens performed in *Drosophila* and zebrafish MD models.

Disease Model	Animal Model	Type of Performed Screen	Identified Drug/Genetic Modifier Mode of Action	Enhanced/Suppressed Phenotype	Ref.
**DMD**	*Drosophila*	Genetic interactors of Dys/Dg	Interactors involved in: muscle, motor & cystoskeleton function, neuronal migration or PCP genes, Notch signaling, TGF-β signaling, EGFR signaling	Wing-vein phenotype (anterior and posterior cross veins detached/altered cross veins)	[27,84]
			Reduction of *mbl* levels enhance muscle phenotypes Dystrophic flies with reduced wunen exhibit less age-dependent degeneration	Abnormal muscle phenotype	
	Zebrafish	Drug screening on sapje and sapje-like mutants	Fluoxetine	Prevention of membrane fragility, survival promotion	[85]
			Aminophylline, Eprizole, Homochlorcyclizine dihydrochloride, Conessine, Equilin, Pentetic acid, Proscillaridin A, Sildenafil, Crassin acetate, Cerulenin, Prostaglandin	Restoration of normal muscle structure in affected embryos	[86,87,88,89]
		Exon-skipping antisense synthetic oligonucleotides (ASO)	Aminoglycoside antibiotics (ataluren-PTC124)		[32,90]
**DM1**	*Drosophila*	Drug screening on DM1 flies(480 interrupted CTG)	10 suppressor drugs: Non-steroidal anti-inflammatory agents, dopamine receptors and monoamine uptake inhibitors, Na^+^ and Ca^2+^ metabolism, Muscarinic, cholinergic and histamine receptors inhibitors, ...	CUG-induced lethality	[91]
		Genetic modifier screening on DM1 flies (480 interrupted CTG)	Suppressors: cnc, Nurf-38, foi, coro, csk, spinster, ... Enhancers: seven up, viking, cg4589	CUG-induced rough-eye phenotype	
	Zebrafish	Drug testing on DM1 zebrafish model (CUG-repeat expansion zebrafish model)	Kinase inhibitor (Ro 31-8220) examination - additional assay to complement drug screen performed in cell culture	Partial rescue of somite number and length to width ratio of the tail	[92]

Notes: Genetic and drug screens performed in Drosophila and zebrafish models of Duchenne muscular dystrophy (DMD) and myotonic dystrophy type 1 (DM1). Abbreviations: PCP Planar Cell Polarity, Dys Dystrophin, Dg Dystrolycan.

The different therapeutic approaches developed to treat neuromuscular disorders and other diseases are (i) ***gene therapy*** to repair the mutated gene, either using exon skipping in the case of a missplicing mutation or by adeno-associated virus (AAV) mediated gene delivery to restore the normal copy of a gene; (ii) ***cell*** ***therapy*** to repair the affected cells that are degenerating in these types of disease; and (iii) ***drug*** ***therapy*** to treat mainly the consequences of the mutations and to soothe the symptoms [93,94,95,96]. 

These strategies utilize different animal models. The gene and cell therapy approaches are developed mainly using mouse models, whereas major advances have been made using *C. elegans*, *D. melanogaster* and the fish *D. rerio*, due to their compatibility with high-throughput screening (HTS) [26,97]. Drug discovery using mammals can be very costly, and so is often used to test only small sets of compounds [60]. Although cell cultures are more efficient for testing large numbers of molecules, their metabolism may also be completely different from that of a whole organism. Hence, maintaining the physiological contexts in the animals allows a better assessment of the toxicity of the molecules tested.

A variety of small molecules have become available from various academic facilities and companies; for example, Prestwick chemical library (Harvard ICCB) contains 1120 small molecules, composed of 90% marked drugs and 10% bioactive alkaloids or related substances. These safe-for-humans compounds were selected for their high chemical and pharmacological diversity [86]. Low molecular weight compounds bind to specific proteins and alter their function, resulting in phenotype changes.

The use of *Drosophila* in the screening of compound libraries helped to find potential therapeutic targets for several neurodegenerative disease models including fragile X syndrome (FXS) [66], Alzheimer’s disease (AD) [98], fragile X-associated tremor/ataxia syndrome (FXTAS) [99] and more recently, Huntington disease (HD) [100].

Regarding muscular dystrophies, Garcia-Lopez and co-workers used screening on their DM1 *Drosophila* model to identify modifiers of CUG-induced lethality. Ten suppressor drugs involved in sodium and calcium metabolism were identified and found to improve survival of DM1 flies [91].

The same DM1 model has also been used to identify drugs that can eliminate the CUG-RNA hairpin formation responsible for the sequestration of MBNL1 and splicing defects. Garcia-Lopez and co-workers [101] applied a positional scanning synthetic combinatorial library (PS-SCL) of d-amino acid hexapeptides, and identified a peptide that binds to CUG repeats and suppresses their toxicity. This ABP1 peptide was also able to reverse missplicing in a DM1 mouse model. The discovery of ABP1 as a suppressor of RNA toxicity in both *Drosophila* and mouse DM1 models supports the view that *Drosophila* is an accurate model system for developing therapeutics in muscular dystrophies.

*Drosophila* is also well-suited for the identification of genetic modifiers, and in particular suppressors of pathological phenotypes [102]. Genetic modifier screens have been performed on *Drosophila* models of spinobulbar muscular atrophy (SBMA) [64], spinal muscular atrophy [103], inclusion body myopathy with early-onset Paget disease and frontotemporal dementia (IBMPFD) [104], and in MD models such as OPMD [57], DM1 [91] and DMD [84]. Regarding DMD, two important genetic interactors have been identified: muscleblind (Mbl) and wunen. The splicing factor Mbl, known to interact with Dg-Dys complex, was found to enhance muscle phenotypes observed in *dys* mutants. By contrast, wunen, a human lipid phosphate phosphatase (LPP) 3 homolog, was identified as a suppressor of the dystrophic wing vein phenotype. The wunen-based suppression of *dys* mutant phenotypes could be related to an increase in the bioactive lipid sphingosine 1-phosphate (S1P) known to promote cell proliferation and differentiation in many tissues, including muscle. The identification of wunen as a suppressor of DMD-associated muscle degeneration could lead to new DMD therapeutic strategies by elevating S1P signalling to suppress or alleviate the dystrophic muscle symptoms [27]. Further genetic modifier screening performed in ageing dystrophic *Drosophila* muscles identified interactors of Dys and/or Dg that are involved in stress response pathways and encode proteins involved in communication between muscle and neuron [105]. Likewise, screening was performed in a DM1 model to identify genetic modifiers of the rough eye phenotype, generated by eye-targeted expression of toxic CUG repeats. Garcia-Lopez and co-workers identified 15 genetic modifiers of eye degeneration, among which genes encoding cell adhesion proteins, regulators of gene expression or cell proliferation and actin cytoskeleton components [91]. Moreover, in recent screening for modifiers of eye and muscle degeneration in DM1 model, a genetic interactor of CUGBP1, Smaug (SMAD4A) was identified and found to act as a suppressor of CUG-induced myopathy [106].

The zebrafish, because of its advantages discussed above, and in particular the ability of zebrafish embryos to absorb chemicals, has become a model of choice in screening and testing new drugs [29,30,31,32,61,92]. This is the case for *sapje* and *sapje-*like dystrophin mutants, which are excellent models for DMD therapeutic screens [85,86,87,88]. For example, Kawahara and co-workers [87], using easy birefringence assays, have identified seven small molecules that influence muscle phenotypes in *sapje* mutants. Although chemical treatment did not restore dystrophin expression, three of the seven compounds restored normal birefringence and showed increased survival of mutant embryos. Among them, aminophylline showed the greatest ability to restore normal muscle structure in affected embryos. This non-selective phosphodiesterase inhibitor (traditionally used for asthma treatment) increases intracellular cAMP levels, causing activation of the cAMP-dependent protein kinase PKA pathway [87]. Aminophylline-treated fish showed increased levels of phosphorylated PKA, indicating PKA-dependent restoration of muscle structure [86]. Other drug screening carried out on *dys* mutants [89] revealed that some aminoglycoside antibiotics (e.g., ataluren-PTC124) can cause read-through of premature stop codon caused by nonsense mutations. Ataluren treatment led to significant improvement of muscle structure and function in *sapje* mutants accompanied by reexpression of dystrophin. 

Another therapeutic strategy involving the zebrafish model is based on the exon-skipping strategy and administration of antisense synthetic oligonucleotides (ASO). Exon skipping partially restores the disrupted reading frame, leading to production of a shorter but still functional dystrophin protein. Statistical and systematic evaluation of exon-skipping in dystrophin-deficient zebrafish mutants demonstrated that the restored *dystrophin* transcripts represent 10%–20% of the normal *dystrophin* transcript level, leading to only partial restoration of muscle function, whereas at least 30%–40% is required to recover dystrophic pathology [63,90]. Altogether, the examples presented above illustrate the importance of the zebrafish model in designing and testing therapeutic approaches for muscular dystrophies and in particular for DMD. 

## 4. Conclusions

Despite an increasing body of breakthroughs made in the understanding of MDs, the molecular mechanisms underlying many muscle disorders remain poorly understood, limiting the development of efficacious therapies. Technological advances, such as next-generation sequencing (NGS), allow the fast identification of gene variants and pathological mutations in MD patients, while simple animal models such as fruitfly and zebrafish provide means to evaluate their significance. Zebrafish and *Drosophila* models have been successfully used in the past to uncover cellular and molecular pathways underlying human muscular diseases, reviewed in [3,30,32,107], revealing particular strengths of each model. For example, the zebrafish model, owing to embryo transparency and visual assessment of muscle structure and mobility phenotypes, is well-suited to large-scale drug screening [29,32,87]. The *Drosophila* model, for which there are large collections of mutants, RNAi lines and other genetic tools, has demonstrated its valuable role in identifying suppressors or enhancers of disease phenotypes through genetic screening [91,97,98].

Overall, this review highlights the role of the zebrafish and *Drosophila* models in understanding the genetic and molecular basis of human MDs and helping to design therapeutic strategies. As these are simple non-mammalian animal models, the knowledge gained needs appropriate validation before it is applied to treating MD in humans.
